# miR-196a-5p promotes metastasis of colorectal cancer via targeting IκBα

**DOI:** 10.1186/s12885-018-5245-1

**Published:** 2019-01-08

**Authors:** He Xin, Chuanzhuo Wang, Zhaoyu Liu

**Affiliations:** Department of Radiology, Shengjing Hospital of China Medical University, 36 Sanhao Street, Shenyang, 110004 People’s Republic of China

**Keywords:** miR-196a-5p, IκBα, Colorectal cancer, Epithelial-mesenchymal transition, Metastasis

## Abstract

**Background:**

MicroRNA-196a-5p (miR-196a-5p) has been reported to be involved in the metastatic process of several cancers. In present work, we aimed to investigate the effects of miR-196a-5p and its potential target IκBα on migration, invasion and epithelial-mesenchymal transition (EMT) of colorectal cancer (CRC) cells.

**Methods:**

CCK-8 assay, wound healing assay and cell invasion assay were performed to evaluate the cell proliferation, migration and invasion. In vivo metastasis models were used to investigate the tumor metastasis ability. Real-time PCR, immunofluorescence staining or western blot were utilized to detect the expression of miR-196a-5p, IκBα, p-IκBα, nuclear p65 and EMT markers including E-cadherin, N-cadherin and fibronectin. Dual luciferase reporter assay was carried out to determine whether there is a direct interaction between miR-196a-5p and IκBα mRNA.

**Results:**

Using SW480 cell with miR-196-5p over-expressed plus SW620 and HCT116 cells with miR-196a-5p knockdown, we found that miR-196a-5p promoted cell proliferation, migration and invasion in vitro and facilitated liver metastasis in vivo. We also observed that miR-196a-5p knockdown or NF-κB pathway inhibition up-regulated E-cadherin while down-regulated N-cadherin and fibronectin. By contrast, miR-196a-5p over-expression promoted EMT process of CRC. Data of dual luciferase reporter assay indicated that miR-196a-5p targeted the IκBα. Moreover, miR-196a-5p down-regulated IκBα expression while up-regulated nuclear p65 expression. Additionally, over-expression of IκBα in CRC cells attenuated the effects of miR-196a-5p on cell migration, invasion and EMT.

**Conclusions:**

miR-196a-5p may play a key role in EMT, invasion and metastasis of CRC cells via targeting the IκBα.

**Electronic supplementary material:**

The online version of this article (10.1186/s12885-018-5245-1) contains supplementary material, which is available to authorized users.

## Background

Colorectal cancer is one of the most common malignant tumors in the gastrointestinal tract, and there are about 1.2 million new CRC cases have been reported worldwide every year [1]. During the last decade, the mortality of CRC had reduced by more than 20% because of the advance in diagnosis and management of CRC [2]. Unfortunately, CRC still causes more than 600,000 deaths per year [1]. The high mortality of CRC is closely correlated with the tumor metastasis, especially the liver metastasis [3]. It has been reported that nearly 25% CRC patients were with metastatic disease at the time of initially diagnosed and approximately half of them will ultimately develop the metastases [4, 5]. With the objective of improving the overall survival of CRC patients, it is urgent to seek new biomarkers in the metastatic CRC and find the interventions.

The microRNAs (miRNAs) are a class of small non-coding RNAs with about 22 nucleotides length [6]. A growing number of studies confirmed that miRNAs played an essential role during the progression of cancer via regulating gene expression by a direct interaction with the 3′ untranslated regions of the targeted mRNAs [7]. For instance, miR-4775 was a risk factor for CRC metastasis and it promoted the invasion, metastasis and EMT of CRC cells by activation of the Smad7/TGFβ signaling cascade [8]. Strikingly, one clinical study showed that miR-196a-5p was in a significantly higher level than the normal adjacent colorectal mucosa and its high expression was associated with the lymph node metastasis and poor prognosis of the CRC patients [9]. In addition, an in vitro study demonstrated that miR-196a promoted CRC cells proliferation and prevented the apoptosis [10]. The above researches indicated that miR-196a-5p was participated in the pathological process of colorectal cancer, while its role and potential regulatory mechanisms in CRC metastasis need to be elucidated.

NF-κB proteins are transcriptional factors which binding to the target genes to regulate their expression [11]. Activation of NF-κB pathway has been reported to be involved in the metastasis of different type of cancers recently. One study from Wang et al indicated that STX2 formed a positive signaling loop with NF-κB that STX2 enhance its own expression by activating the NF-κB pathway and ultimately facilitate the CRC metastasis [12]. Moreover, it has been elucidated that deactivation of the NF-κB signaling pathway by Lipocalin2 suppressed the invasion, metastasis and EMT of the CRC cells [13]. These studies inspired us that activation of the NF-κB pathway may make great contribution to the metastatic process of CRC.

In present work, we employed the SW480 cells with miR-196-5p over-expressed plus SW620 and HCT116 cells with miR-196a-5p knockdown to explore the role of miR-196a-5p on CRC migration, invasion and liver metastasis. We further investigated whether miR-196a-5p exert its pro-metastatic effects via facilitating the EMT by a direct inhibition of the IκBα.

## Methods

### Cell culture

The human colorectal cancer (CRC) cell lines of SW480, SW620 and HCT116 were obtained from Shanghai Zhong Qiao Xin Zhou Biotechnology Co., Ltd. and cultured in DMEM medium (Gibco, USA) supplemented with 10% fetal bovine serum (BI, USA) at 37 °C in 5%CO_2_. In order to evaluate the influence of NF-κB pathway on the migration, invasion and EMT of CRC cells, cells were treated with NF-κB pathway inhibitor BAY 11–7082 (10 μM, medchemexpress, USA) for 48 h before following experiments.

### IκBα plasmid construction and cell transfection

In order to construct the IκBα expression plasmid, the full-length cDNA of IκBα was amplified and inserted into pcDNA3.1 vector with HindIII and BamHI sites. Lipofectamine 2000 (Invitrogen, USA) was employed to transfect IκBα plasmid into SW480 cells. Further, miR-196a-5p mimics, NC mimics, miR-196a-5p inhibitor and NC inhibitor purchased from Shanghai GenePharma Co., Ltd. were transfected into CRC cells using the Lipofectamine® RNAiMAX (Invitrogen, USA) following the manufacturer’s protocol.

### Lentivirus infection

The lentiviral (LV) particles that over-expressing miR-196a-5p (LV-miR-196a-5p) and its negative control (LV-NC) or silencing miR-196a-5p (LV-anti miR-196a-5p) and its negative control (LV-anti NC) were purchased from Wanleibio Co., Ltd. CRC cells were seeded in 6-well plates at a density of 4 × 10^5^/well. 24 h latter, the lentivirus was added into the culture medium to infect the CRC cells. After screening with Purine toxin (Solarbio, China), CRC cells with stable miR-196a-5p over-expressed or knockdown were obtained.

### Dual luciferase reporter assay

The 3′-UTR region of wide-type of IκBα was amplified and inserted into pmirGLO plasmid at Nhe I and Sal I sites. Furthermore, miR-196a-5p mimics or the miR-196a-5p mutant was cotransfected with Luc-IκBα or the control into HEK 293 T cells using the Lipofectamine 2000 (Invitrogen, USA). Luciferase assays were carried out with the Dual-Luciferase Reporter Assay System (Promega, USA) referring to the instructions.

### Real-time PCR

The total RNA from CRC cells in different conditions was extracted using the TRIpure kit (BioTeke, China). After quantization of concentration, RNA was reversely transcribed into cDNA by the Super M-MLV reverse transcriptase (BioTeke). Quantitative PCR was carried out to determine the expression levels of miR-196a-5p and IκBα using 2 × Power Taq PCR MasterMix (BioTeke) and SYBR Green (Solarbio, China). The procedure for IκBα was as follows: first cycle including 94 °C for 5 min, 94 °C for 10 s, 60 °C for 20 s, 72 °C for 30 s, and then 40 cycles of 72 °C for 2 min 30 s, 40 °C for 1 min 30 s, melting from 60 °C to 94 °C for 1 s every 1 °C. While the miR-196a-5p expression level was measured under following condition: 94 °C for 2 min, 94 °C for 15 s, 60 °C for 15 s, 72 °C for 15 s followed with 40 cycles of 72 °C for 2 min 30 s, 40 °C for 5 min 30 s, melting from 60 °C to 94 °C for 1 s every 1 °C. The data were calculated by 2^-ΔΔCt^ or 2^-ΔCt^ method and sequences information was shown in Table 1.

**Table 1:** Primers used in Real-time PCR

Name	Sequences
hsa-miR-196a-5p	F: CCGACGTAGGTAGTTTCATGTT
	R: GTGCAGGGTCCGAGGTATTC
	RT:GTTGGCTCTGGTGCAGGGTCCGAGGTATTCGCACCAGAGCCAACCCCAAC
hsa-U6	F: CTCGCTTCGGCAGCACA
	R: AACGCTTCACGAATTTGCGT
	RT:GTTGGCTCTGGTGCAGGGTCCGAGGTATTCGCACCAGAGCCAACAAAATATGG
IκBα	F: CATCCTGAAGGCTACCAACTAC
	R: CATCAGCACCCAAGGACAC
β-actin	F: CTTAGTTGCGTTACACCCTTTCTTG
	R: CTGTCACCTTCACCGTTCCAGTTT

*F* Forward, *R* Reserve

### CCK-8 assay

CRC cells were seeded into 96-well plates at 3 × 10^3^/well. The cell viability was measured at 12 h, 24 h, 48 h and 72 h after transfection. CCK-8 regent (KeyGEN BioTECH, China) was added into culture medium 10 μl per well. After 1 h incubation, the optical density of solution was measured using a microplate reader at 450 nm.

### Wound healing assay

The transfected or infected CRC cells were cultured in serum-free DMEM containing mitomycin C (Sigma, USA) for 1 h. Subsequently, a sterile 200 μl pipette tip was employed to cause scratches. Representative images of cells migrating into wounds were taken at 0 h, 24 h or 12 h after the scratch.

### Cell invasion assay

Cell invasion assay was performed on 24-well transwell chambers coated with Matrigel (BD, USA). In brief, the upper chamber was added into 200 μl DMEM containing 1 × 10^4^ CRC cells and the lower chambers were filled with 800 μl culture medium containing 30% FBS. After 24 h incubation, migrated cells were fixed with 4% paraformaldehyde for 20 min and stained with the crystal violet (Amresco, USA) for 5 min. Cells on lower surface were counted with an inverted microscope.

### In vivo tumor metastasis assay

To assess the effects of miR-196a-5p on tumor metastasis, we employed 4-week-old BALB/c nude mice to establish in vivo metastasis models. The thymic aplasia of the nude mice results in immunodeficiency and could avoid interference from host immune system. The nude mice used in present study were obtained from Beijing HFK Bioscience Co., Ltd. (Beijing, China; License number: SCXK (Jing) 2014–0004) and housed in a standard laboratory environment (12 h day-night cycle; temperature: 25 ± 1 °C; humidity: 50 ± 5%). During the experiments, mice were free access to the water and food and no adverse events were observed. In present work, 24 mice were randomly divided into four groups: SW480-LV-NC group (*n* = 6); SW480-LV-miR-196a-5p group (n = 6); HCT116-LV-anti NC group (n = 6); HCT116-LV-anti miR-196a-5p group (n = 6). According to the experimental group dividing, 2 × 10^6^ transfected CRC cells were injected into spleens of the anesthetized nude mice and the spleens were resected 48 h latter. All mice were sacrificed 7 weeks after the injection and the hepatic metastases have been evaluated.

### H&E staining

The fixed liver tissues were embedded in paraffin followed with slicing into 5 μm sections. After dewaxing, the sections were stained with hematoxylin (Solarbio, China) and eosin (Sangon, China). The metastatic foci were observed under the OLYMPUS DP73 microscope.

### Western blot assay

CRC cells or liver metastatic tissues were lysed with RIPA Lysis Buffer (Beyotime, China) containing 1% PMSF (Beyotime). Besides, the nuclear protein was isolated by the Nuclear and Cytoplasmic Protein Extraction Kit (Beyotime). Protein from different conditions were separated by 6–14% SDS-PAGE and then transferred to PVDF membranes. After blocking with 5% non-fat milk or 1% BSA, the membranes were incubated with following primary antibodies at 4 °C overnight: anti-E-cadherin (1:1000, 60,335–1-Ig, Proteintech, China), anti-N-cadherin (1:1000, 66,219–1-Ig, Proteintech, China), anti-fibronectin (1:500, 15,613–1-AP, Proteintech, China), anti-p-IκBα (1:500, bs-2513R, Bioss, China), anti-IκBα (1:500, bs-1287R, Bioss, China), anti-p65 (1:500, 10,745–1-AP, Proteintech, China), anti-β-actin (1:500, KGAA001, KeyGen, China) and Histone H3 (1:2000, AM8433, ABGENT, USA). After rinsed with TSBT, the membranes were incubated with their corresponding secondary antibodies at 37 °C for 45 min. The protein bands were visualized using the ECL reagent (Beyotime).

### Immunofluorescence staining

The CRC cells were seeded on slides and then fixed in 4% paraformaldehyde for 15 min. After permeabilized with 0.1% tritonX-100 (Beyotime, China) for 30 min at room temperature, the slides were blocked with goat serum (Solarbio, China). Subsequently, cells were incubated with antibodies of E-cadherin (1:100, 60,335–1-Ig, Proteintech, China), N-cadherin (1:100, 66,219–1-Ig, Proteintech, China), Fibronectin (1:50, 15,613–1-AP, Proteintech, China) overnight at 4 °C. Then CRC cells were incubated with the goat anti-rabbit Cy3-conjugated IgG (1:400, A0516, Beyotime) or goat anti-mouse Cy3-conjugated IgG (1:400, A0521, Beyotime). Lastly, cells were counterstained with DAPI (Sigma, USA) before capturing images using the fluorescence microscope (Olympus, Japan).

### Statistical analysis

The data were presented as mean ± SD and were obtained from at least three individual experiments. One-way or two-way ANOVA test followed with post hoc Bonferroni’s test were used to analyze data. While the statistical difference between two groups were analyzed by student t test. Correlations were analyzed using Pearson’s correlation test. A *P* < 0.05 was seen as statistically significant.

## Results

### miR-196a-5p promoted the cell proliferation of CRC cells

**Fig. 1:**
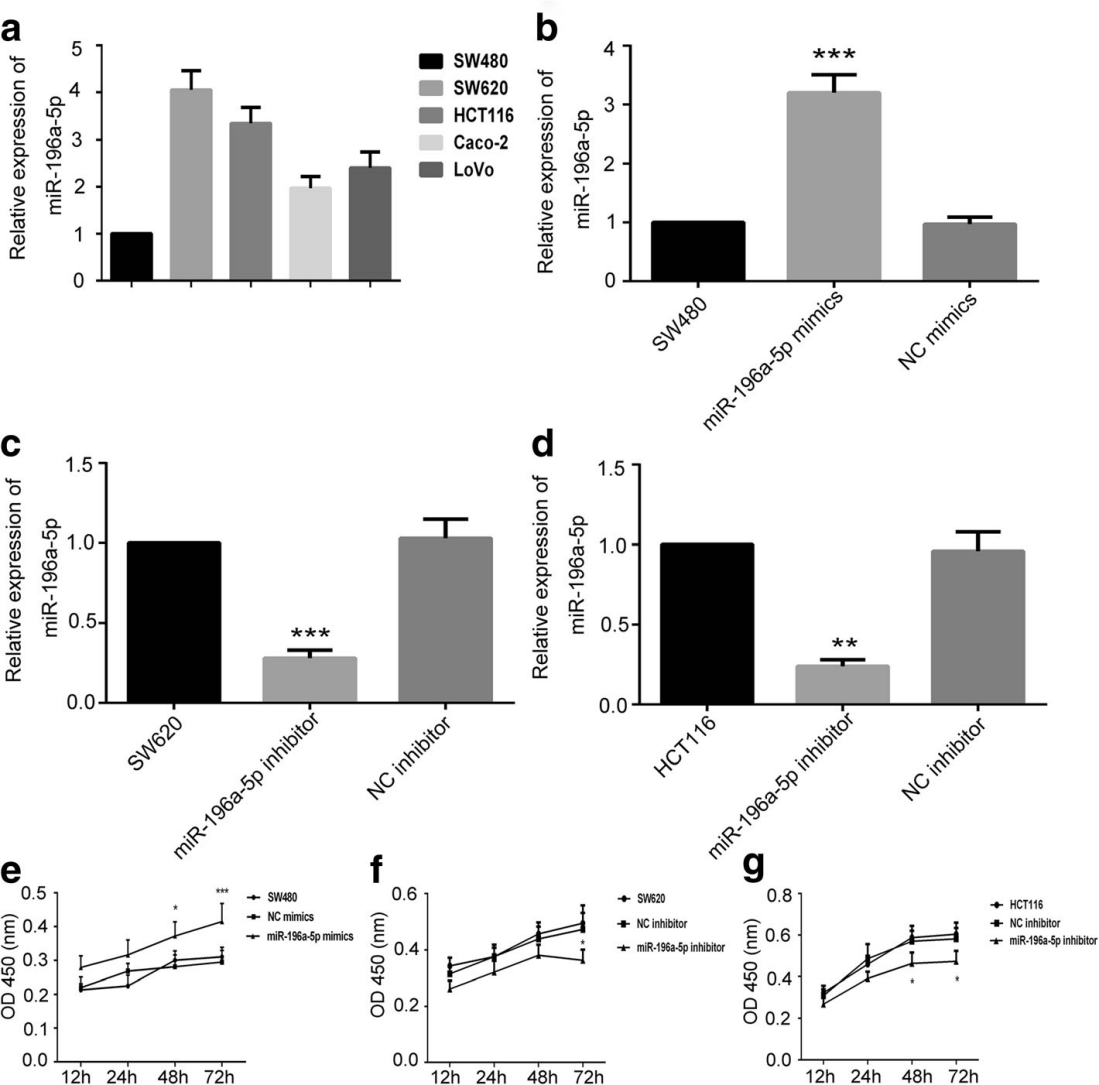
The effects of miR-196a-5p on cell proliferation. **a** The expression level of miR-196a-5p in CRC cell lines. The expression of miR-196a-5p in **b** SW480, **c** SW620 and **d** HCT116 cells after transfection were measured by Real-time PCR. Cell viability of transfected **e** SW480, **f** SW620 and **g** HCT116 cells were determined by CCK-8 assay. Data were shown as mean ± SD, ***P < 0.001, **P < 0.01 and *P < 0.05 compared to NC group

We firstly performed Real-time PCR to detect the expression level of miR-196a-5p in various CRC cell lines. As shown in Fig. 1a, the primary colon cell SW480 revealed a lowest expression of miR-196a-5p, while the metastatic colon cell SW620 depicted a 4.05-fold elevation of miR-196a-5p than SW480. We also observed that miR-196a-5p was highly expression in HCT116 cell while weak expression in Caco-2 and LoVo cell lines.

We then assessed whether the expression level of miR-196a-5p in CRC cells influence their proliferation. With this objective, we used miR-196a-5p mimic and inhibitor to induce or silence the miR-196a-5p expression in CRC cells (Fig. 1b, c and d). The results of CCK-8 demonstrated that over-expression of miR-196a-5p significantly accelerated the cell proliferation, while the cell viability was lower in miR-196a-5p-silenced cells than the control (Fig. 1e, f and g).

### miR-196a-5p facilitated the migration and invasion of CRC cells

**Fig. 2:**
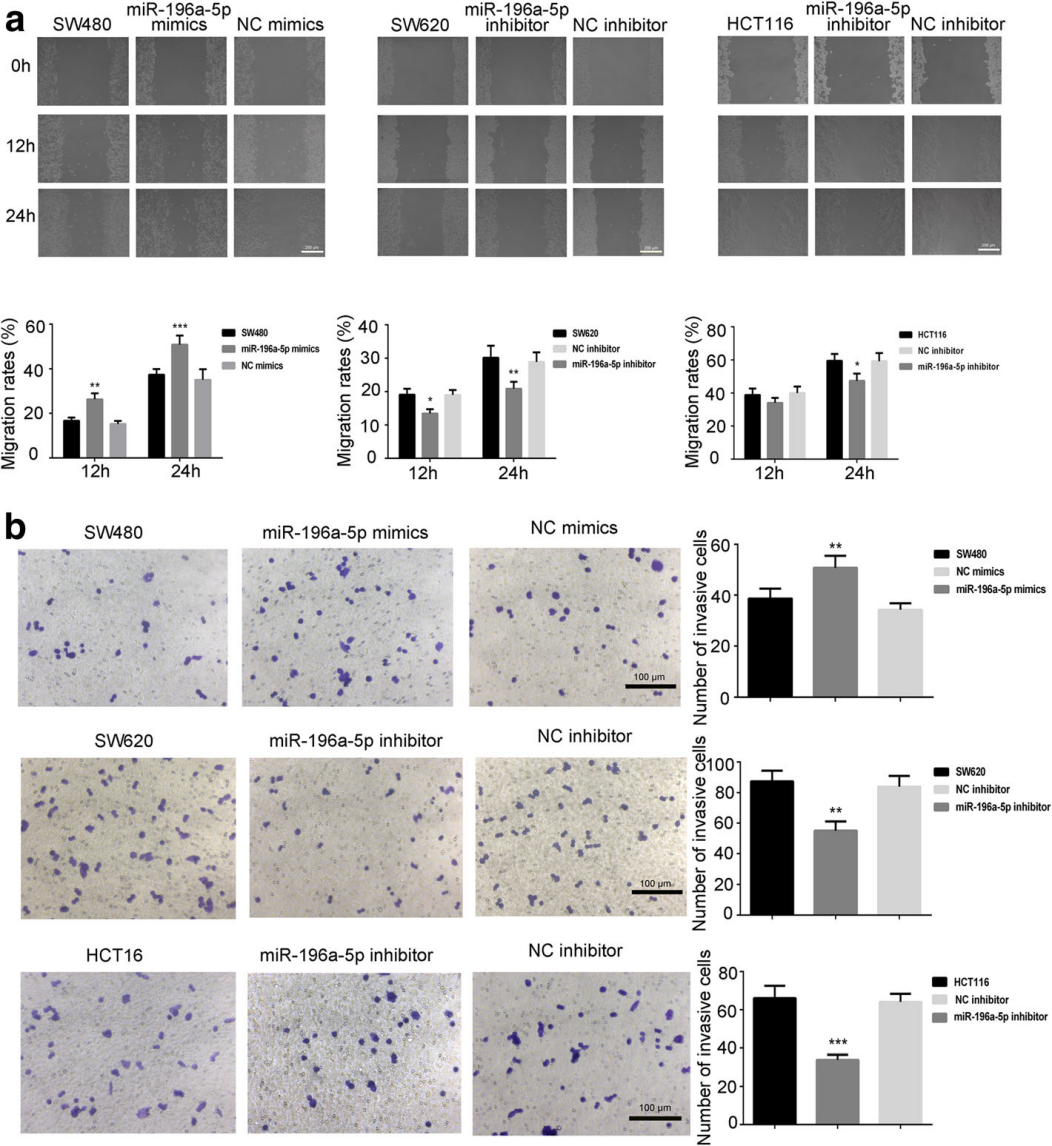
The effects of miR-196a-5p on cell migration and invasion. **a** The representative images and analysis of wound healing assay in transfected CRC cells. **b** The representative images and analysis of trans-well invasion assay in CRC cells. Data were shown as mean ± SD, ***P < 0.001, **P < 0.01 and *P < 0.05 compared to NC group

We subsequently investigated the influence of miR-196a-5p on the cell mobility. Data from wound healing assay indicated that over-expression of miR-196a-5p in SW480 cells enhanced the migration rate to 26.30 ± 2.70% and 50.83 ± 4.11% at 12 and 24 h, respectively (Fig. 2a). We also observed that knockdown of miR-196a-5p in SW620 and HCT116 cells decreased the migration rates at 24 h (Fig. 2a). Interestingly, The SW480 cell migration at 24 h was higher than that of SW620 cells [14, 15]. We further found that over-expression of miR-196a-5p in SW480 cells increased the number of invading cells by 31%, while knockdown of miR-196a-5p reduced the invading cells by 35–55% in SW620 and HCT116 cells (Fig. 2b).

### miR-196a-5p aggravated the liver metastasis in vivo

**Fig. 3:**
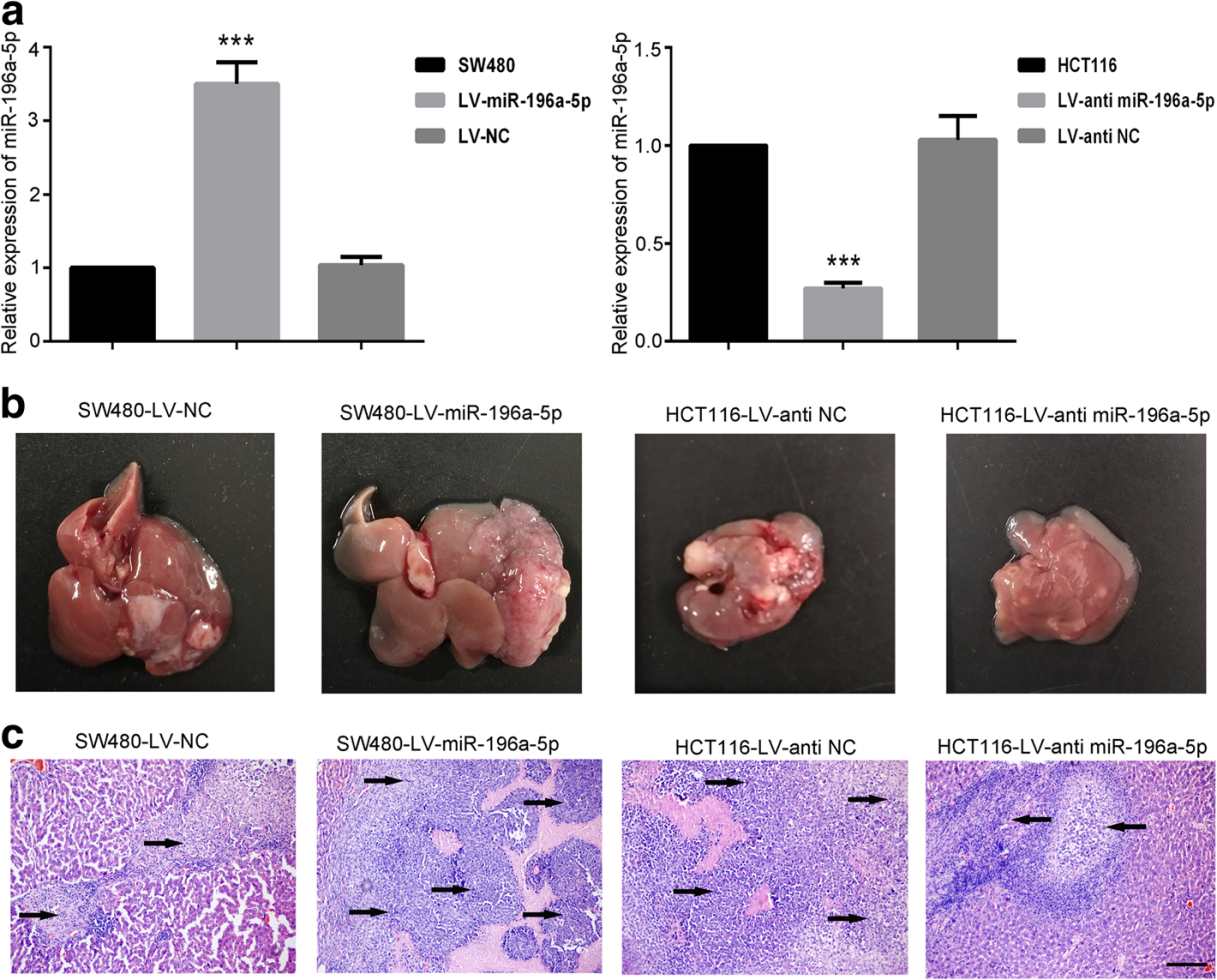
The effects of miR-196a-5p on the liver metastasis of CRC. **a** The expression level of miR-196a-5p in infected CRC cells. **b** The representative images of macroscopic metastasis in the liver from mice injected CRC cells. **c** H&E staining indicated the metastatic foci identified in the liver of mice after injection of CRC cells. Scalar bar: 200 μm. Data were shown as mean ± SD, ***P < 0.001, compared to NC group

The effects of miR-196a-5p on liver metastasis were also been evaluated in our present study. We firstly established SW480 cells stably over-expressed miR-196a-5p, and HCT-116 cells with miR-196a-5p knockdown (Fig. 3a). After injection these CRC cells into spleens for seven weeks, laparotomy was conducted. We observed that SW480 cells which over-expressed miR-196a-5p induced more metastatic nodules formation in liver, while knockdown of miR-196a-5p in HCT-116 cells attenuated the tumor metastasis (Fig. 3b). As shown in Fig. 3c, histologic analyses further verified that injection with miR-196a-5p-expressed CRC cells increased the number of micrometastatic lesions in liver, whereas injection of miR-196a-5p knockdown cells had opposite effects. The western blot results showed that the expression of IκBα and E-cadherin was decreased, and N-cadherin was increased in liver metastatic nodules induced by miR-196a-5p overexpressing cells (Additional file 1a). Conversely, we found that IκBα and E-cadherin protein levels were increased, and N-cadherin protein levels were decreased in liver metastatic nodules induced by miR-196a-5p downregulated cells (Additional file 1b).

### miR-196a-5p promoted the epithelial-mesenchymal transition of CRC cells

**Fig. 4:**
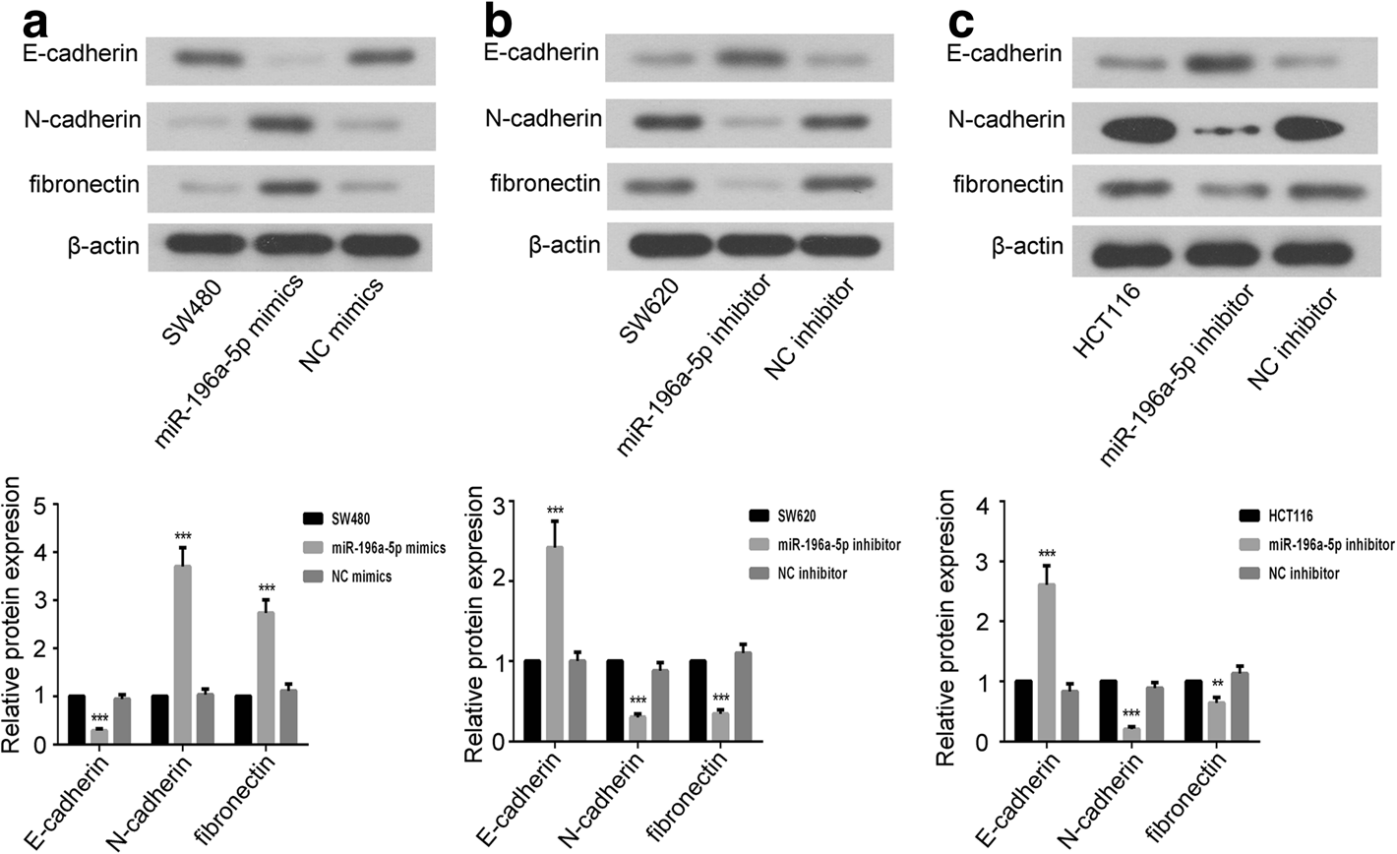
The effects of miR-196a-5p on the expression of EMT-related proteins in CRC cells. The representative images and analysis of western blot for E-cadherin, N-cadherin, fibronectin in **(a)** SW480, **(b)** SW620 and **(c)** HCT116 cells. Data were shown as mean ± SD, ***P < 0.001 and **P < 0.01 compared to NC group

**Fig. 5:**
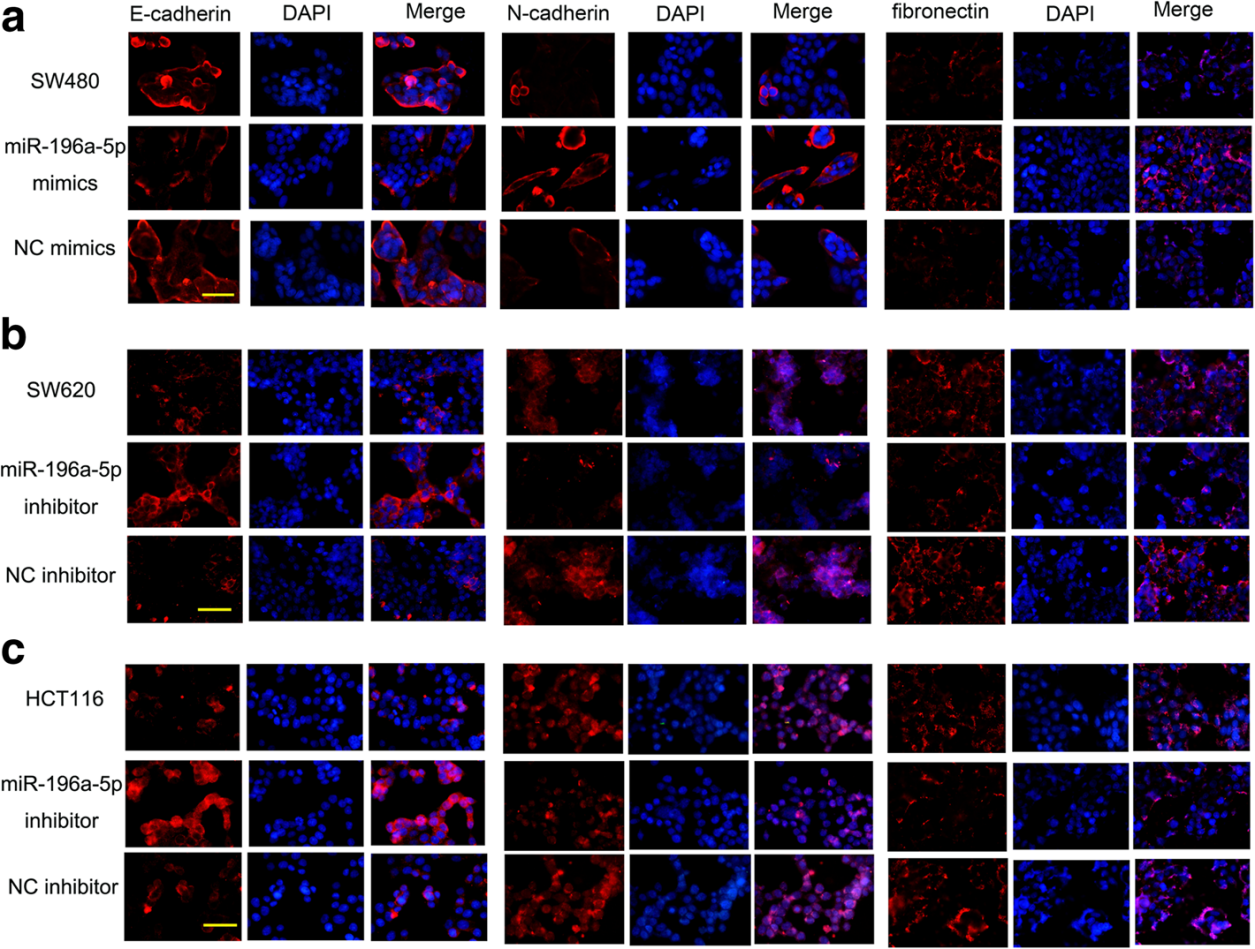
The influence of miR-196a-5p on the EMT process of CRC cells. Immunofluorescence staining showed the expression of EMT-related markers in **a** SW480, **b** SW620 and **c** HCT116 cells after transfected with miR-196a-5p mimic or miR-196a-5p inhibitor. Scalar bar: 50 μm

Western blot assay and immunofluorescence staining were performed to evaluate the effects of miR-196a-5p on the expression of epithelial markers E-cadherin and mesenchymal markers N-cadherin and fibronectin. As shown in Fig. 4 and Fig. 5, over-expression of miR-196a-5p down-regulated the E-cadherin, whereas up-regulated the N-cadherin and fibronectin. Contrastingly, E-cadherin was increased while N-cadherin and fibronectin were decreased in miR-196a-5p knockdown CRC cells.

### Inhibition of NF-κB signaling pathway suppressed the EMT of CRC cells

**Fig. 6:**
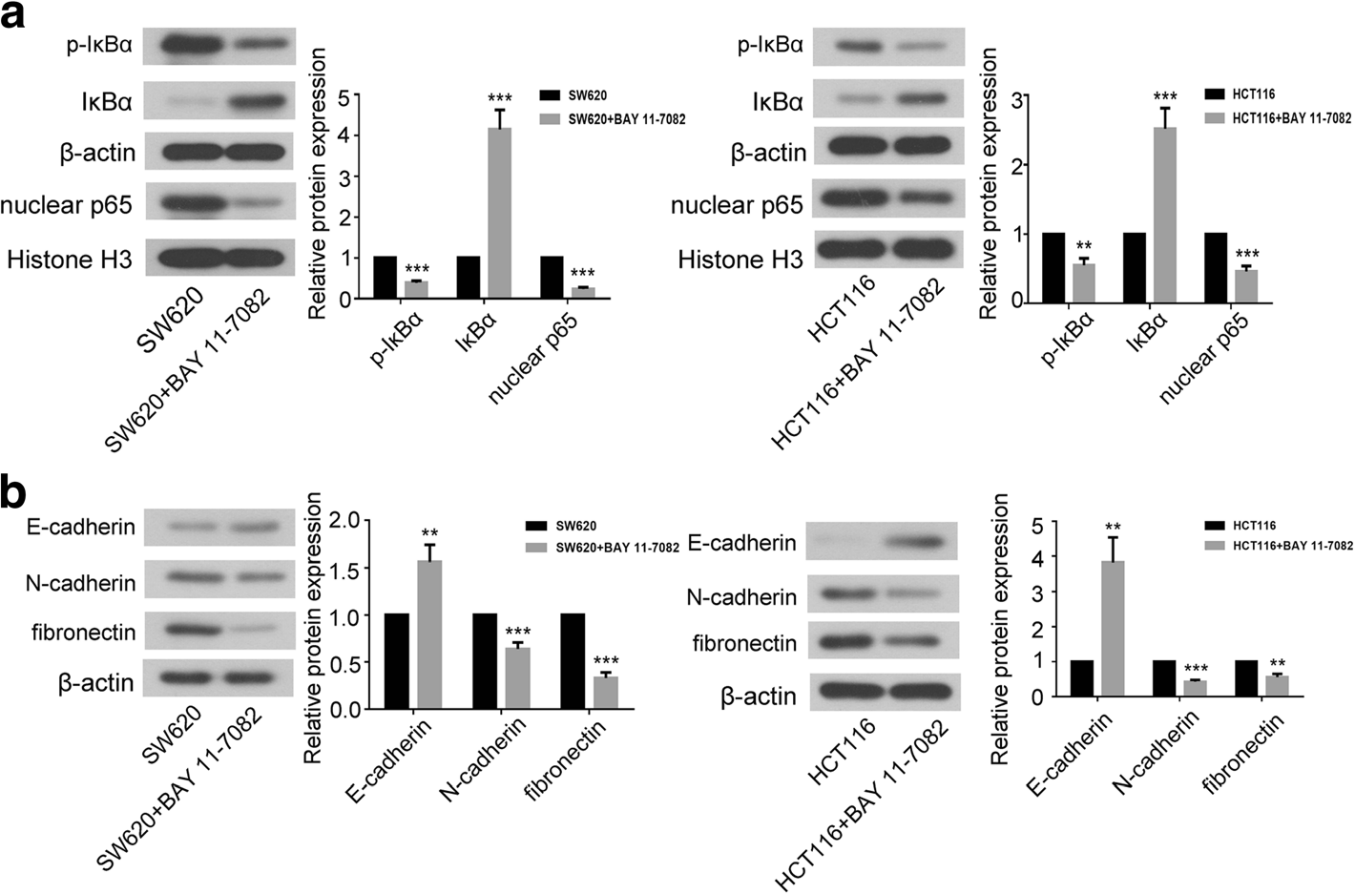
The effects of NF-κB signaling pathway on EMT. **a** The representative images and analysis of western blot for IκBα、p-IκBα and nuclear p65 in CRC cells treated with BAY 11–7082 (inhibitor of NF-κB pathway). **b** The expression levels of E-cadherin, N-cadherin and fibronectin in SW620 and HCT116 cells were measured by western blot. Data were shown as mean ± SD, ***P < 0.001 and **P < 0.01 compared to non-treated group

We employed BAY 11–7082, the inhibitor of NF-κB signaling pathway, to investigate the role of NF-κB pathway in EMT. As shown in Fig. 6a, BAY 11–7082 decreased the phosphorylation level of IκBα and nuclear expression of p65 in SW620 and HCT116 cells. BAY 11–7082 up-regulated the total expression level of IκBα in CRC cells. Furthermore, BAY 11–7082 increased the expression of E-cadherin whereas decreased the expression of N-cadherin and fibronectin in both SW620 and HCT116 cells (Fig. 6b).

### miR-196a-5p promoted EMT and mobility of CRC cells via targeting IκBα

**Fig. 7:**
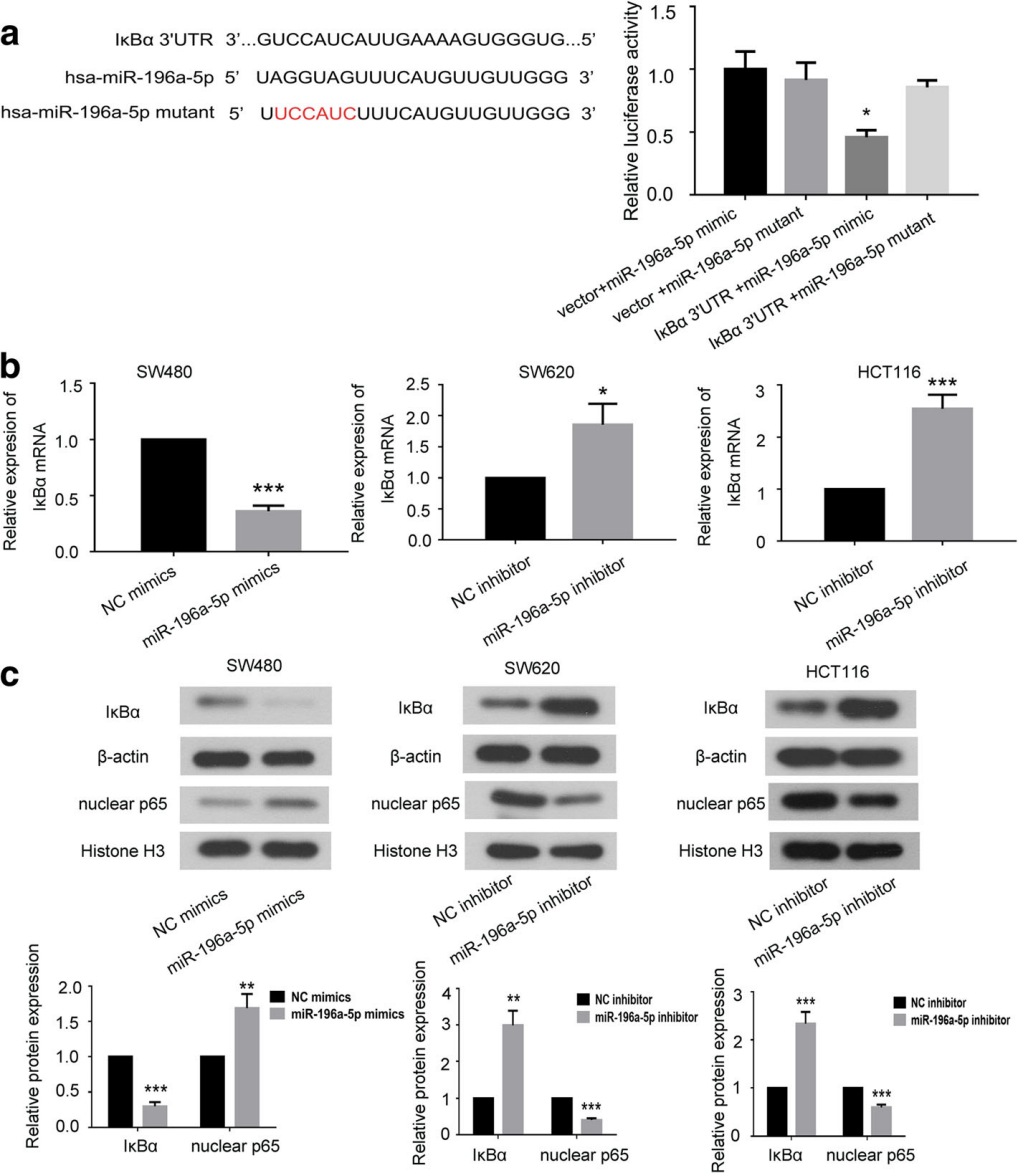
IκBα was a target of the miR-196a-5p. **a** The predicted seed region of IκBα bound to miR-196a-5p and the mutated sequence used in present work was shown at left. The luciferase activity was measured at 48 h after HEK293 cells cotransfected with vector/miR-196a-5p mimic, IκBα-WT/miR-196a-5p mimic or IκBα-WT/miR-196a-5p-mutant plasmid. **b** The expression of IκBα mRNA in CRC cells after transfection. **c** The effects of miR-196a-5p on the expression of IκBα and nuclear p65 were determined by western blot. Data were shown as mean ± SD, ***P < 0.001, **P < 0.01 and *P < 0.05 compared to NC group

**Fig. 8:**
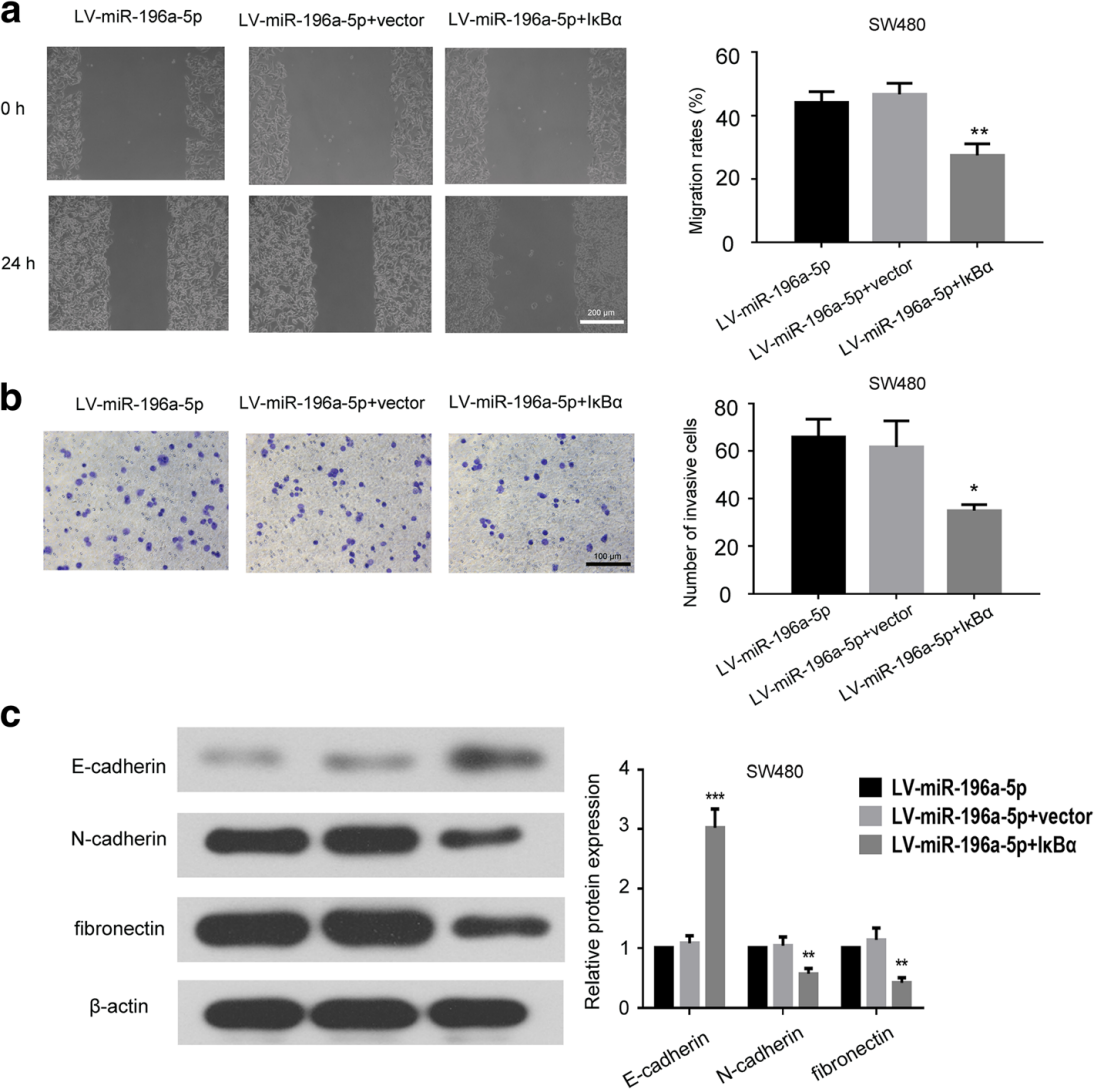
miR-196a-5p promoted the migration, invasion and EMT of CRC cells via targeting IκBα. **a** The representative images and analysis of wound healing assay in CRC cells after transfection. **b** The representative images and analysis of trans-well invasion assay in CRC cells. **c** The expression level of EMT-related proteins in transfected CRC cells were determined by western blot. ***P < 0.001, **P < 0.01 and *P < 0.05 compared to LV-miR-196a-5p + vector group

By analyzing the database of microrna.org, we found that IκBα may be a downstream target of the miR-196a-5p. The expression of IκBα and its correlation with miR-196a-5p were evaluated in CRC cells (Additional file 2). The result showed a negative correlation tendency between miR196 and IκBα expression in CRC cells (*r* = − 0.372), but no significance (*p* = 0.54). We performed dual luciferase reporter assay to determine whether there was an interaction between IκBα mRNA and miR-196a-5p. As shown in Fig. 7a, miR-196a-5p mimic decreased the luciferase activity of reporter containing 3′UTR of IκBα, while miR-196a-5p mutant did not have similar effects. We found that induced the expression of miR-196a-5p down-regulated the IκBα both in mRNA and protein level, whereas miR-196a-5p knockdown had opposite effects (Fig. 7b and c). We further observed that over-expression of miR-196a-5p increased the nuclear p65 level, and knockdown of miR-196a-5p decreased the expression of nuclear p65 (Fig. 7c). Moreover, we employed IκBα plasmid to induce the expression of IκBα in SW480 cells (Additional file 3). As shown in Fig. 8a and b, inducing the IκBα expression by transfection of IκBα plasmid significantly decreased the migration rate and number of invading cells in miR-196a-5p overexpressing SW480 cells. Further, over-expression of IκBα in miR-196a-5p overexpressing CRC cells increased E-cadherin levels and inhibited N-cadherin and fibronectin levels (Fig. 8c).

## Discussion

Tumor metastasis seriously affects the prognosis of CRC patients [16]. While several studies indicated that miR-196a-5p was involved in the pathological process of CRC, the clear regulatory mechanisms of miR-196a-5p in CRC metastatic process have yet to be elucidated. The merit of our present work was firstly indicated that miR-196a-5p may target the IκBα to regulate the EMT process and finally promoted CRC cells invasion and metastasis.

The miR-196a-5p was initially identified as an oncogenic miRNA which direct targeted annexin A1 to promote proliferation and prevent apoptosis of esophageal cancer cells [17]. With further investigations, researchers found that miR-196a-5p was aberrant expression in various cancer tissues such as cervical cancer and non-small cell lung cancer, and its expression level was correlated with the progression of these cancers [18, 19]. For instance, miR-196a-5p was significantly over-expressed in the oral cancer tissues and it accelerated the cell migration and invasion of oral cancer cells through the NME4-JNK-TIMP1-MMP axis [20]. Intriguingly, one study from Schimanski [21] and his colleagues demonstrated that miR-196a-5p was up-regulated in CRC samples and it played a pro-oncogenic role by reinforcing CRC cells invasion and their sensitivity to the platinum derivatives. They also observed that over-expression of miR-196a-5p enhanced the lung metastases of CRC in vivo. In line with these, our work demonstrated that over-expression of miR-196a-5p promoted the proliferation, migration, invasion and the liver metastasis of CRC cells, indicating that hyper proliferated CRC cells by miR196 may show an increase in cell migration and liver metastasis, while miR-196a-5p knockdown attenuated the cell viability, invasion and metastasis of CRC cells. These results confirmed the oncogenic effects of miR-196a-5p, especial the pro-metastatic effects.

Epithelial-mesenchymal transition is a reversible process which is been considered as a critical program during the metastatic cascade in several types of cancers including the CRC [22, 23]. During this process, cells suppressed the E-cadherin and expressed the mesenchymal proteins such as N-cadherin, fibronectin to loss the cell-cell adhesion and acquire migratory and invasive abilities [24, 25]. Inspired by these, we evaluated the effects of miR-196a-5p on the EMT process of CRC. As expected, inducing expression of miR-196a-5p in CRC cells promoted the EMT by down-regulating E-cadherin while up-regulating N-cadherin and fibronectin, whereas silencing of miR-196a-5p exerted opposite effects. The above investigations suggested that promoting the EMT contributed a lot to the pro-metastatic effects of miR-196a-5p in CRC.

It is worth mentioning that several literatures demonstrated that IκBα may work as a downstream target of miR-196a-5p during cancer progression. One study from Yang et al showed that miR-196a took an oncogenic role in glioblastoma multiforme by targeting IκBα [26]. The IκBα is a negative regulator of the NF-κB signaling pathway. After being phosphorylated and degraded by stimulation, IκBα lose the bind with NF-κB and let NF-κB translocate to nucleus to activate the target genes [27, 28]. Moreover, it has been showed that suppressing the phosphorylation of IκBα may partially inhibit the invasion and EMT of gastric cancer cells [29]. In non-small cell lung cancer cells, preventing the phosphorylation of IκBα and the subsequently activation of NF-κB was benefit for suppressing the EMT process [30].

Consideration of above observations, we speculated miR-196a-5p may also facilitate the EMT of CRC via regulating the IκBα expression. By performing the dual luciferase reporter assay, we verified that miR-196a-5p directly bound to the IκBα gene. We also investigated the effects of miR-196a-5p on the expression of IκBα pathway. As expected, miR-196a-5p over-expression decreased the expression of IκBα and increased the level of nuclear p65, while miR-196a-5p knockdown up-regulated the IκBα and down-regulated the nuclear p65 in CRC cells. The data suggested that IκBα may be a target of the miR-196a-5p in CRC cells.

We further evaluated that whether inhibition of the NF-κB pathway attenuated the EMT of CRC. The results demonstrated that deactivation of NF-κB pathway increased epithelial markers E-cadherin but decreased the mesenchymal markers N-cadherin and fibronectin. As IκBα is the natural inhibitor of NF-κB signaling pathway, these findings implied that miR-196a-5p and IκBα may take opposite roles in the EMT of CRC. Additionally, over-expression of IκBα in CRC cells attenuated miR-196a-5p induced-migration, invasion and EMT of CRC cells, which further confirmed that miR-196a-5p exerted its pro-metastatic effects by facilitating the EMT process via targeting IκBα. In order to further clarify the pro-metastatic effects and the potential mechanisms of miR-196a-5p in CRC, we will perform clinical studies to analyze the correlation between miR-196a-5p levels and liver metastasis. The relationship between the expression of miR-196a-5p and IκBα in the clinical CRC tissues will also be investigated in future.

## Conclusions

In conclusion, our data demonstrates that miR-196a-5p facilitates the proliferation, metastasis and invasion of CRC cells. Additionally, the pro-metastatic property of miR-196a-5p is exerted through regulating the EMT process via targeting the IκBα. Our findings suggest that regulating miR-196a-5p may be a potential strategy to ameliorate the tumor metastasis of CRC.

## Additional files

Additional file 1: Western blot assay was employed to evaluate the expression levels of IκBα, E-cadherin and N-cadherin in liver metastatic nodules induced by (a) miR-196a-5p overexpressing or (b) miR-196a-5p downregulated CRC cells. ***P < 0.001 compared to LV-NC or LV-anti-NC group. (TIF 1803 kb)

Additional file 2: (a) The mRNA level of IκBα in CRC cell lines. (b) The correlation between miR-196a-5p and IκBα mRNA levels in CRC cells. (TIF 718 kb)

Additional file 3: Transfection of IκBα plasmid induced the expression of IκBα in SW480 cells. (a) The protein level of IκBα in SW480 cells. (b) The mRNA level of IκBα in SW480 cells. ***P < 0.001 compared to vector group. (TIF 1284 kb)

## Abbreviations

CRC: Colorectal cancer
EMT: Epithelial-mesenchymal transition
miRNAs: microRNAs
