# Supplementary figures and images for: miR-196a-5p promotes metastasis of colorectal cancer via targeting IκBα
Source: BMC Cancer. 2019 Jan 8;19:30. doi: 10.1186/s12885-018-5245-1 (PMC6325824; doi:10.1186/s12885-018-5245-1)

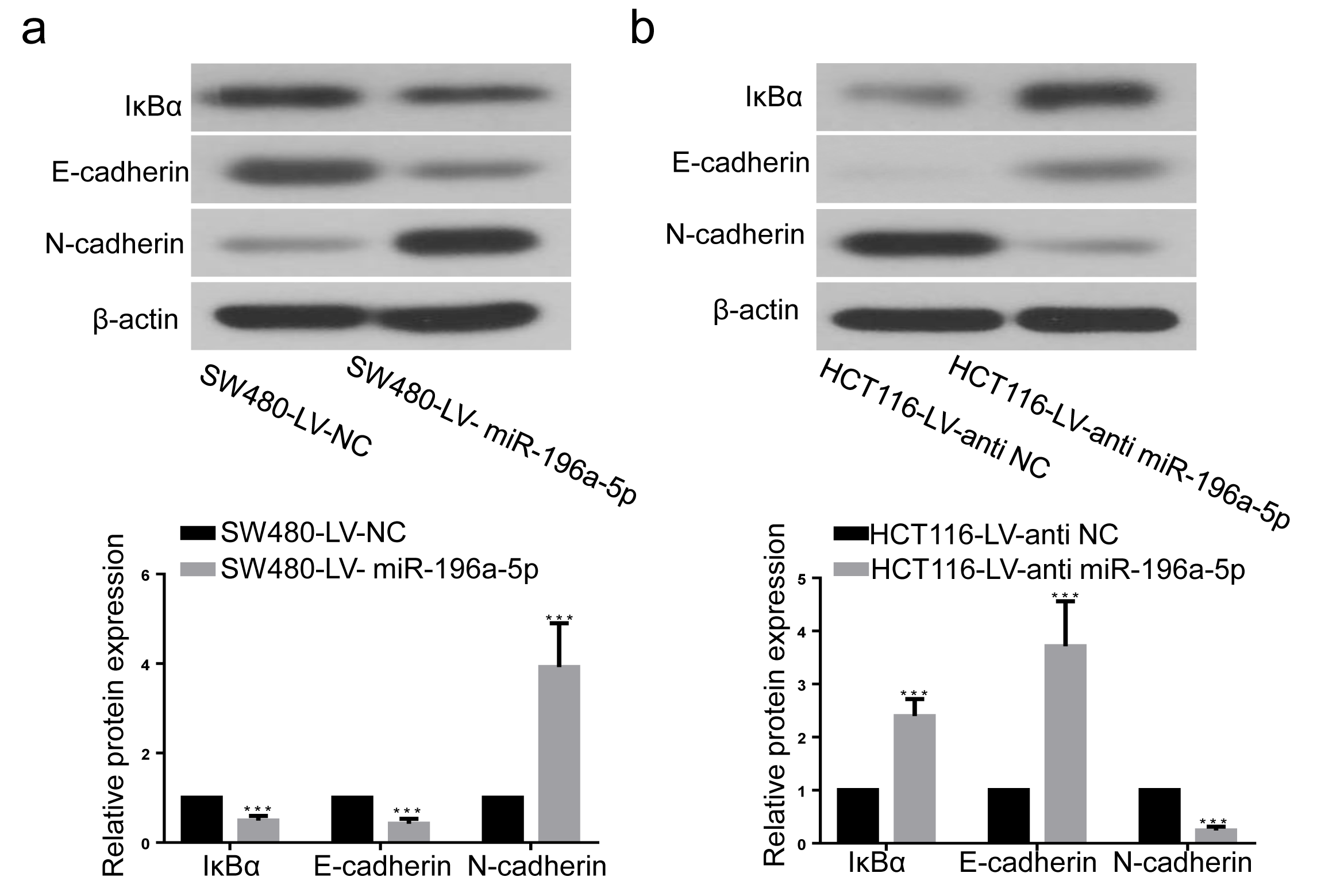

Supplement: Supplementary file 1 — Western blot assay was employed to evaluate the expression levels of IκBα, E-cadherin and N-cadherin in liver metastatic nodules induced by (a) miR-196a-5p overexpressing or (b) miR-196a-5p downregulated CRC cells. ***P < 0.001 compared to LV-NC or LV-anti-NC group. (TIF 1803 kb) [file 12885_2018_5245_MOESM1_ESM.tif]

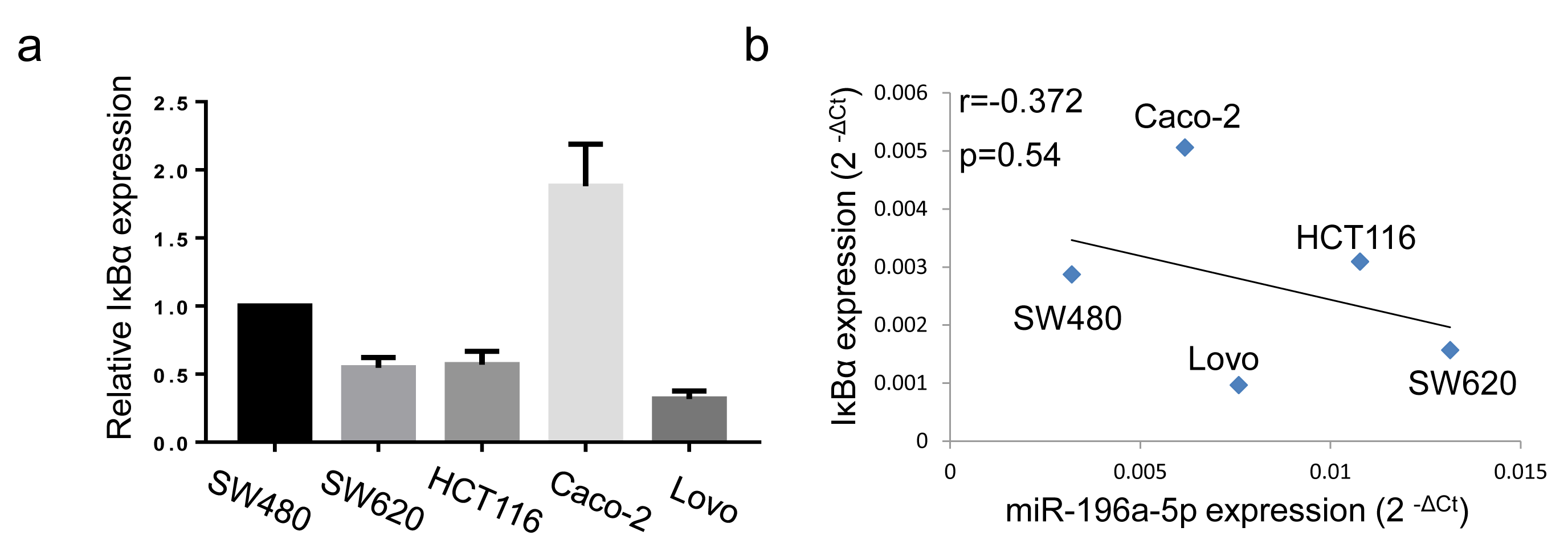

Supplement: Supplementary file 2 — (a) The mRNA level of IκBα in CRC cell lines. (b) The correlation between miR-196a-5p and IκBα mRNA levels in CRC cells. (TIF 718 kb) [file 12885_2018_5245_MOESM2_ESM.tif]

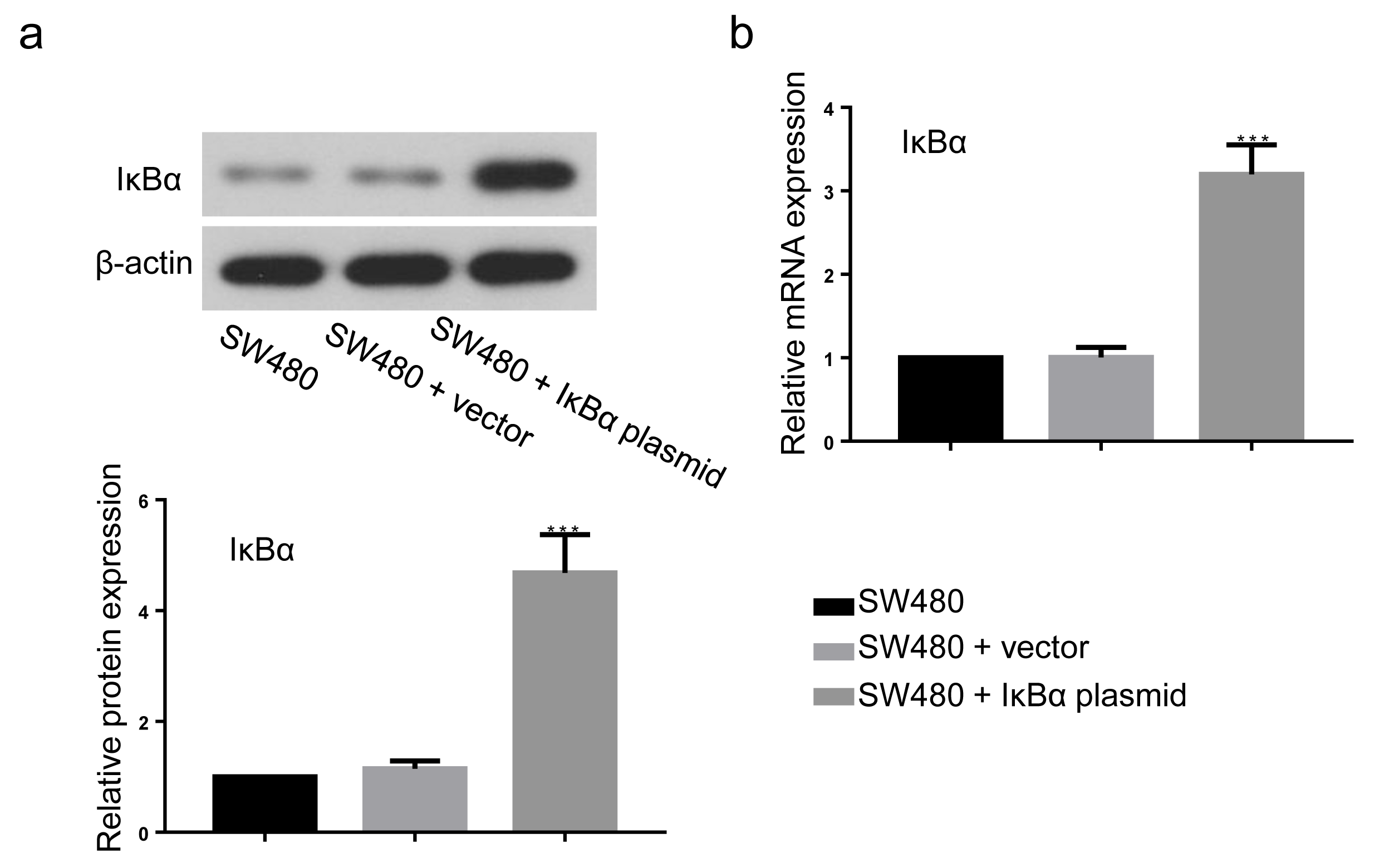

Supplement: Supplementary file 3 — Transfection of IκBα plasmid induced the expression of IκBα in SW480 cells. (a) The protein level of IκBα in SW480 cells. (b) The mRNA level of IκBα in SW480 cells. ***P < 0.001 compared to vector group. (TIF 1284 kb) [file 12885_2018_5245_MOESM3_ESM.tif]
